# Effects of Graphene Oxide (GO) and Reduced Graphene Oxide (rGO) on Green-Emitting Conjugated Copolymer’s Optical and Laser Properties Using Simulation and Experimental Studies

**DOI:** 10.3390/polym15234572

**Published:** 2023-11-29

**Authors:** Saradh Prasad, Raya H. Alhandel, Nassar N. Asemi, Mohamad S. AlSalhi

**Affiliations:** Research Chair on Laser Diagnosis of Cancers, Department of Physics and Astronomy, College of Science, P.O. Box 2455, King Saud University, Riyadh 11451, Saudi Arabianassarasemi@gmail.com (N.N.A.); malsalhi@ksu.edu.sa (M.S.A.)

**Keywords:** conjugated copolymer, TD-DFT simulation, Amplified spontaneous emission (ASE), reduced graphene oxide, time-resolved spectroscopy (TRS), fluorescence quenching

## Abstract

The properties of a conjugated copolymer (CP), poly[(9,9-Dioctyl-2,7-divinylenefluorenylene)-alt-co-(2-methoxy-5-(2-ethylhexyloxy)-1,4-phenylene) (PDVF-co-MEH-PV), were investigated in the presence of graphene oxide (GO) and reduced graphene oxide (rGO) using absorption, fluorescence, laser, and time-resolved spectroscopy. CPs are usually dissolved in low-polar solvents. Although GO does not dissolve well, rGO and PDVF-co-MEH-PV dissolve in chloroform due to their oxygen acceptor sites. Hence, we studied rGO/PDVF-co-MEH-PV (CP/rGO), performing all experiments and simulations in chloroform. We performed simulations on PDVF-co-MEH-PV, approximate GO, and rGO using time-dependent density-functional theory calculations to comprehend the molecular dynamics and interactions at the molecular level. The simulation polymer used a tail-truncated oligomer model with up to three monomer units. The simulation and experimental results were in agreement. Further, the PDVF-co-MEH-PV exhibited fluorescence, laser quenching, rGO-mediated laser blinking, and spectral broadening effects when GO and rGO concentrations increased. The experimental and simulation results were compared to provide a plausible mechanism of interaction between PDVF-co-MEH-PV and rGO. We observed that for lower concentrations of rGO, the interaction did not considerably decrease the amplified spontaneous emissions of PDVF-co-MEH-PV. However, the fluorescence of PDVF-co-MEH-PV was considerably quenched at higher concentrations of rGO. These results could be helpful for future applications, such as in sensors, solar cells, and optoelectronic device design. To demonstrate the sensor capability of these composites, a paper-based sensor was designed to detect ethanol and nitrotoluene. An instrumentation setup was proposed that is cheap, reusable, and multifunctional.

## 1. Introduction

Researchers have identified a wide range of potential applications in optoelectronics for organic conjugate molecules, including organic light-emitting diodes [1], solar cells [2], and field-effect transistors [3]. There has been a significant focus on utilizing organic materials for laser applications [4,5,6], as these materials offer unique light–matter interactions and flexibility for fabrication through wet-fabrication methods [7]. New advancements in material simulations and synthesis also drive this interest in organic materials in bottom-up fabrication techniques in organic chemistry [8], which enable the precise control of the physical and optoelectronic properties through controlled iterative research and development methodologies. However, achieving a high performance in all essential aspects, such as low threshold energy, stability, and color tuning, remains a challenge for all organic solution-processed devices [9,10,11,12]. The synthesis of GO and rGO also advances, making it much easier to find new applications and opportunities for scaling up [13,14].

Conjugated polymers (CPs) are a class of organic materials that possess alternating double bonds along the polymer chain, allowing for the efficient movement of electrons and holes, making them suitable for optoelectronic devices such as light-emitting diodes (LEDs) [15,16,17] and lasers [18,19]. CPs are often combined with other materials to tailor the properties in hybridizing or compositing processes. Organic–inorganic hybrid light-emitting diodes (HyLEDs) based on ZnO nanoparticle blends used as LED electron injection layers were designed by I. Hamilton et al. [20].

Poly [(9,9-dioctyl-2,7-divinylenefluorenylene)-alt-co-(1,4-phenylene)], one of the derivatives of polyfluorene-co-phenylene, has garnered considerable attention among CPs due to its high quantum yield and excellent thermal stability [21,22,23]. As it is well known, the band gap determines the wavelength of light that conjugated polymers can absorb and emit. PFO, a blue-emitting polymer, has a large band gap, resulting in blue emission at 410 nm and 430 nm. MEH-PPV, a red-emitting polymer, has a significantly lower band gap, resulting in a red emission at 560 nm and 605 nm, and when PFO and MEH-PPV are combined (copolymerization) to modify the band gap of PFO-at-co-MEH-PPV via rearranging the π orbital interaction of the two monomer units, it results in a green emission at 505 nm [24,25].

Incorporating graphene oxide (GO) and reduced graphene oxide (rGO) into conjugated copolymers can enhance the optoelectronic properties of the resulting composite materials through several mechanisms. First, graphene and rGO have high electrical conductivity, which can improve the charge transport properties of composite materials. In particular, poly(3-hexylthiophene)/rGO (P3HT/rGO) was used to design OFET and volatile organic compounds (VOCs) (Cheon, H.J., et al. [26]). Second, silver nanowires (AgNWs)/P3HT/rGO can act as an efficient conductive material with conductivities between 15 and 42 S/m [27]. Third, the chemical functionalities on the surface of rGO can interact with the conjugated polymer chain, leading to improved stability and reduced degradation of the composite materials, such as MEH-PPV/rGO [28]. rGO/thiophenic composites were proposed as a promising material for photovoltaic applications [29]. Various CP/GO and rGO nanocomposite materials have been explored as materials for applications such as energy storage, catalysis, and biomedical applications [30]. 

The presence of rGO has been shown to alter the optical properties of CPs, particularly fluorescence and amplified spontaneous emissions (ASEs). The quenching of fluorescence and ASEs is an important mechanism when a fluorophore interacts with a quencher. However, the effects of rGO on the ASE characteristics of CPs were not studied or understood well, let alone PDVF-co-MEH-PV. Hence, GO and rGO may be useful in constructing EO devices by injecting or expelling electrical charges. Fluorescence, laser, and time-resolved spectroscopy were not used in previous research on the fluorescence quenching (FQ) behavior of GO and rGO on PDVF-co-MEH-PV. As a result, we were eager to investigate PDVF-co-MEH-PV composites in ethylene glycol and chloroform, which include both GO and rGO. This research examined how GO and rGO resulted in FQ and enhanced absorption in PDVF-co-MEH-PV. The quantum yield and fluorescence intensity of PDVF-co-MEH-PV decreased with increasing concentrations of GO and rGO in the solution. We examined the connection between the spectrum and the spatial broadening of rGO/PDVF-co-MEH-PV mirrorless lasing capabilities. Surprisingly, we discovered that rGO did not negatively affect the ASE performance of PDVF-co-MEH-PV when used in small doses. As expected, at more significant quantities of rGO, the ASEs of the CP were disrupted. The time-resolved spectroscopy (TRS) investigation demonstrated ASE blinking on timescales below a nanosecond. PDVF-co-MEH-PV/GO and PDVF-co-MEH-PV/rGO composites in solution media can be reported here for the first time for chloroform. Thin films of TPDVF-co-MEH-PV/rGO are simple to produce and can aid in the study and development of optoelectronic devices.

## 2. Materials and Methods 

GO and chloroform were bought from Sigma-Aldrich (USA); they were exfoliated and a few layers thick. A moderately modified variety of the G. Compagini et al. method involves using a nanosecond pulse (3rd and 2nd harmonic of Nd:YAG, 10 Hz, 5 ns pulse) to reduce GO [31]. The synthesis of rGO involved irradiating graphene oxide in deionized water with the 2nd and 3rd harmonics (532 nm and 355 nm, 5 ns pulse, 10 Hz repetition rate) of an Nd:YAG laser. G. Compagini et al. used only 532 nm, but we used both 355 nm and 532 nm in our synthesis procedure. The ablation threshold is projected to be 150 mJ/cm^2^ and diverse photon fluences were used for experiments oscillating between 150 and 850 mJ (maximum peak power of 170 MW). At low fluence values below 150 mJ/cm^2^, water remained unaffected. However, when radiation is kept at elevated levels, gas bubbles may form due to the disintegration of water molecules, which could yield reactive species by photomechanical ablation. Finally, when sufficient energy was applied, layered material appeared to fluctuate randomly at the water/air interface that was then collected on the solid substrate as rGO layers in dispersion. The CP/GO and CP/rGO were sonicated for 5 min to disperse the GO and rGO in the polymer. The CP/GO and rGO samples were prepared by mixing them in chloroform and deionized water to form concentrations of 1, 2, 3, 5, 20, and 30% wt%. We do not discuss the 10 and 15% results, as the results were close to those of 5 and 20%, respectively. We observed considerable changes at low concentrations. Hence, we studied 1, 2, 3, and 5%. Further, the GO or rGO did not dissolve well to form a homogeneous solution in nonpolar solvents (like water), as shown in Figure S1, which shows the dipole moment of the GO (12.19 debye) and rGO (4.66 debyes) simulated using a sketch illustrating that rGo has a four-fold lesser dipole moment; hence, it dissolves well in nonpolar solvents [32] (e.g., chloroform or dichlorobenzene [33]). Similarly, the rGO dissolved relatively less in a nonpolar solvent. However, the combination of chloroform and RGO found a homogeneous solution. Hence, we studied the laser-induced fluorescence and time-resolved spectroscopy of CP/rGO exclusively in chloroform. 

**Figure 1:**
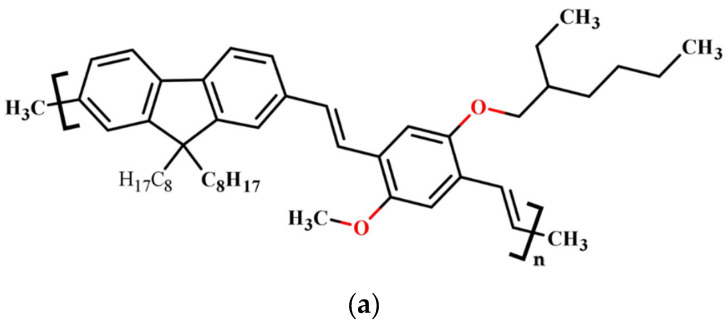

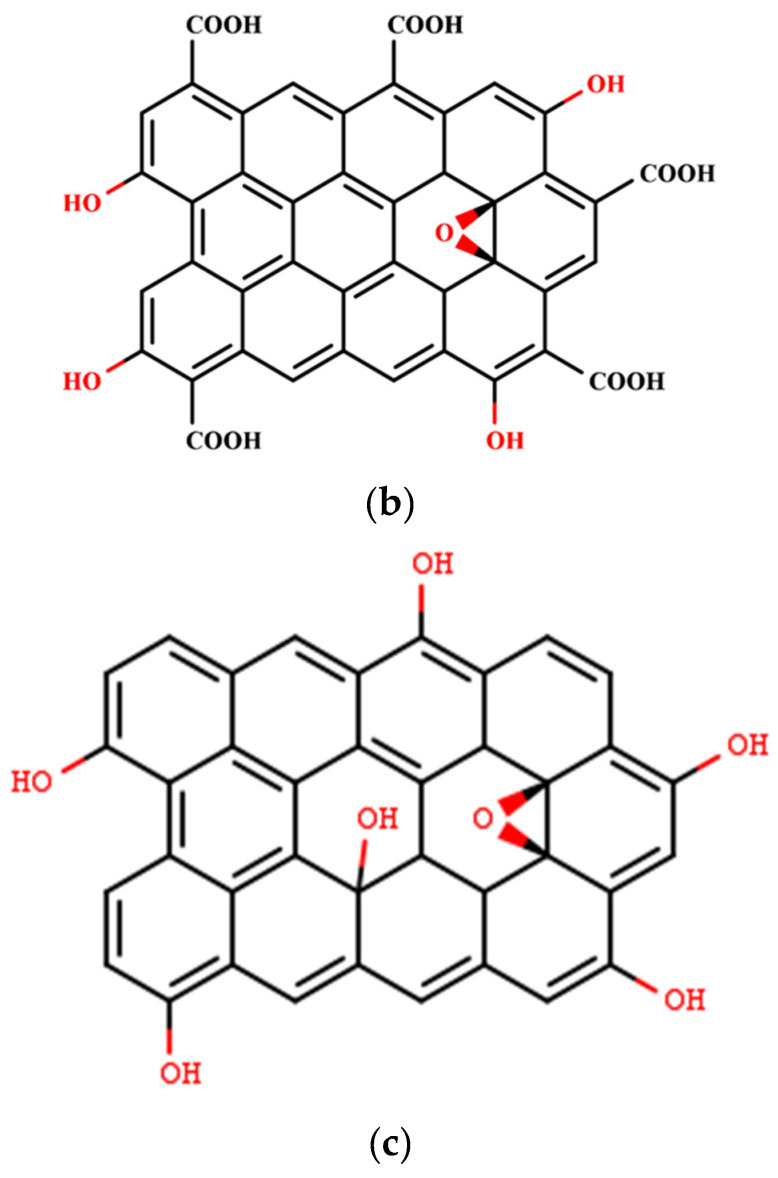
Molecular structure of (**a**) PDVF-co-MEH-PV, (**b**) GO, and (**c**) rGO.

Figure 1 depicts the chemical composition of (a) PDVF-co-MEH-PV, (b) GO, and (c) rGO. The molecular structures of graphene oxide (GO) and reduced graphene oxide (rGO) were simulated using previous studies by K Tadyszak et al. [34] and R Geetha Bai et al. [35]. Identical structures were employed in the simulation of the time-dependent density-functional theory. The absorption spectra were measured using a Perkin Elmer Lambda 950 spectrophotometer located in Llantrisant, United Kingdom. The measurements were taken in the range of 100 to 1100 nm. On the other hand, the fluorescence spectra were measured using a spectrofluorometer (LS 55) from the same company. The measurements were taken at room temperature at 200 to 1000 nm.

A quartz lens with a spherical shape and a focal length of 5 cm was utilized to concentrate the pump pulse from the Nd:YAG laser for longitudinal pumping. The hybrid rGO/PDVF-co-MEH-PV and pure PDVF-co-MEH-PV solutions were stimulated by administering a concentrated pulse in the form of a line. The hybrid solutions exhibited laser-induced fluorescence (LIF) and ASEs at suitable pump energies. The spectrograph used for examination was the Ocean Optics Spectroscopy model USB4000-XR1-ES, located in Maybachstrasse, Ostfildern, Germany. It is equipped with a linear array charge-coupled device that converts light signals into a digital spectrum for subsequent analysis [36].

CP/rGO composites are dropped on a silicon substrate and spun at 500 or 1000 rpm using a CHEMAT KW-4A Spin Coater (Northridge, CA, USA). To make the sensors, CP/rGO was drop-cast on small pieces (20 × 30 mm approximately) of ROCO 80 GSM A4 paper.

The computational analyses were performed at King Saud University using Marvin Sketch, Avogadro, and the Gaussian 16 software suite [26] on a workstation equipped with an AMD Ryzen 9 3950X processor with 16 cores and 32 threads. The workstation also has 128 GB of RAM and a 1 TB M.2 SSD. 

## 3. Results and Discussion

### 3.1. Computational Studies

We simulated the orbital structure of (a) PDVF-co-MEH-PV, (b) approximated GO, and (c) rGO using the time-dependent density-functional theory B3PLY (Becke three-parameter Lee–Yang–Parr) method with a 6–31 (G) basis set in Gaussian 16 (G16) software [37]. We optimized the structure in Avagadro 0.2.0 software and later exported the input file to G16. We calculated many properties using simulations; however, in this study, we included only Frontier Molecular Orbital (FMO) structures [18].

**Figure 2:**
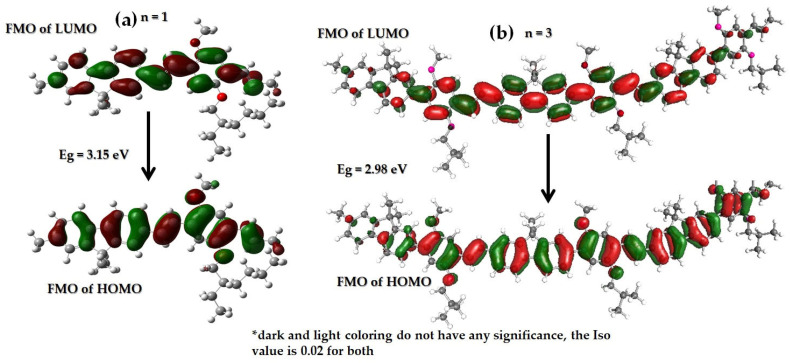
FMO structure of (**a**) full monomer and (**b**) tail-truncated trimer PDVF-co-MEH-PV simulated using the B3PLY method and 6–31 G basis set.

Figure 2a presents the FMO structure of the full monomer PDVF-co-MEH-PV simulated using the B3PLY method and 6–31 G basis set. The highest occupied molecular orbital (HOMO) is mostly vertical along the main block of the PDVF-co-MEH-PV. Additionally, oxygen (O) had an MO for monomers. The lowest unoccupied molecular orbital (LUMO) showed a horizontal FMO. Computing a higher number of monomer (n = 2, 3, and 4) models resulted in a very high calculation burden of tails (PFO leg and MEH horn), and their contribution to FMO was insignificant due to lack of conjugation. Hence, the tail has been curtailed for more extensive PDVF-co-MEH-PV oligomer calculations. Figure 2b presents the FMO structure of the tail-truncated trimer PDVF-co-MEH-PV simulated using the B3PLY method and 6–31 G basis set. The HOMO and LUMO were similar to monomers, but toward the edges (both left and right), the FMO strengths waned compared to that of the PDVF-co-MEH-PV monomer, indicating that the edges of polymers were less active; this also indicates the reduction in the size of the chromophore. The exact boundaries of a chromophore are still debatable. However, the bandgap was comparable to the experimental values at the shape of the FMO.

**Figure 3:**
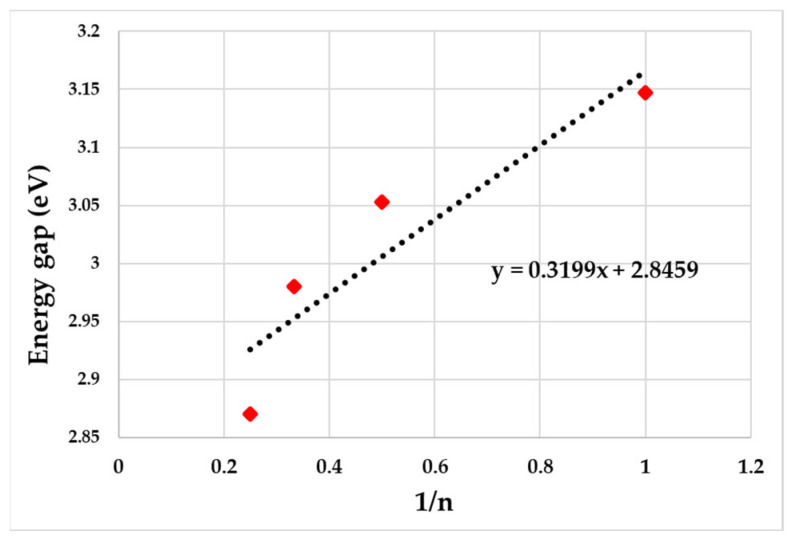
Estimated band gap from simulated energy gap at different monomer units plotted against the inverse of monomer units. The dotted line is fitting of simulated energy gap for n = 1 to 4 and corresponding equation is given inside (y = 0.3199x + 2.8459).

Figure 3 demonstrates the estimation of the band gap from the simulated energy gap at different monomer units plotted against the inverse of monomer units. The band gap of PDVF-co-MEH-PV reduces as the number of monomers increases from 3.15 eV, 3.08 eV, 2.98 eV, and 2.83 eV for repetition units 1, 2, 3, and 4, respectively. The bandgap for the polymer calculated as (1/n) tended towards zero and Eg = 2.846 eV. 

**Figure 4:**
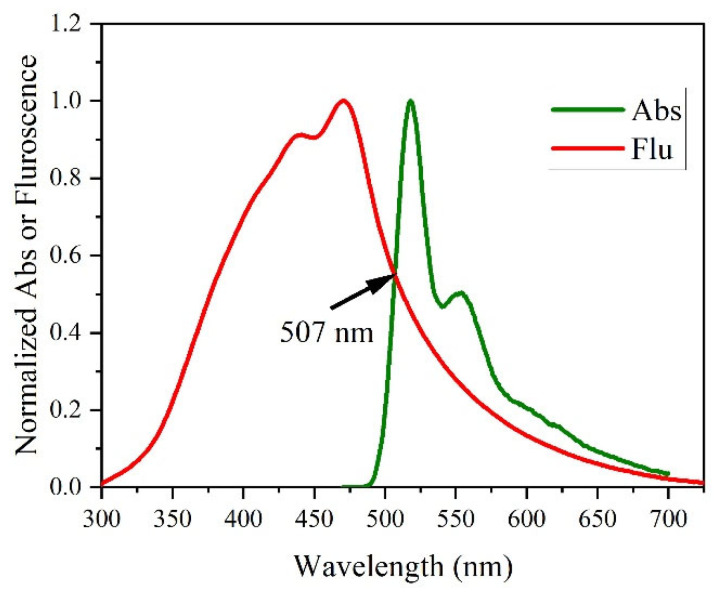
Experimentally estimated band gap using the fluorescence and absorption spectra intersection.

Experimentally, the band gap was estimated using the intersection of fluorescence and absorption spectra method, as shown in Figure 4, and had a band gap of 2.834 eV. When compared to the simulation-based band, the error was only 0.4%. Hence, we can assume that the FMO and other DFT simulations were valid, and interpretations can be made using them. 

**Figure 5:**
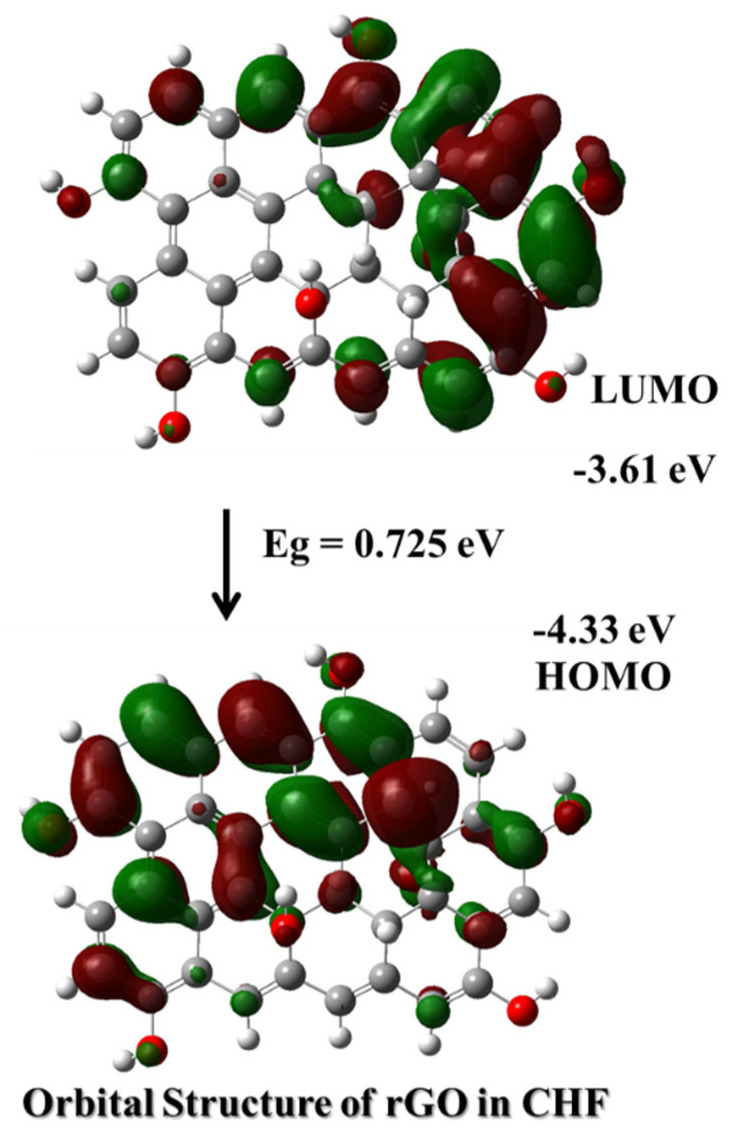
FMO structure of the approximated rGO simulated using the B3PLY method and 6–31 G basis set.

The GO and CP did not form a homogeneous solution; hence, we ran a simulation for GO using a smaller molecular footprint, and the FMOs are given in Figure S2. Figure 5 illustrates the simulated FMOs of rGO (approximated) (also given in Figure S2 for comparison). The direct bandgap was 0.725 eV. However, the first excited state contains other transitions from an excited state to a ground state and vice versa. The involved electronic robotics were HOMO-1 (230), HOMO (231), LUMO (232), and LUMO + 1 (233). The net band gap was 0.116 eV. Supplementary Table S1 shows the vibrational transitions, their contributions, and the oscillator strengths of the first excited state of rGO from the simulation, as illustrated in Figure S3. The rGO simulation illustrated that HOMO spread throughout the structure, and the LUMO was only half strengthened and presented mainly in the inner part of the structure; this indicates that the weak direct radiative transition and the nonradiative solid transition are usual for any size of GO [38,39,40].

Further, the observed proximity in energy levels between the LUMO of PDVF-co-MEH-PV and the HOMO of GO/rGO suggests a feasible pathway for electron transfer, which is a key mechanism in fluorescence quenching. Furthermore, the spatial distribution of these orbitals, especially the localization of LUMO in PDVF-co-MEH-PV and HOMO in GO/rGO, indicates a high probability of overlap upon interaction, facilitating electron transfer [41]. The suggested processes for the fluorescence quenching of rGO are believed to involve Förster resonance energy transfer (FRET) and nonradiative migration or decay of charges [42].

### 3.2. Absorption of GO and rGO in Deionized Water

**Figure 6:**
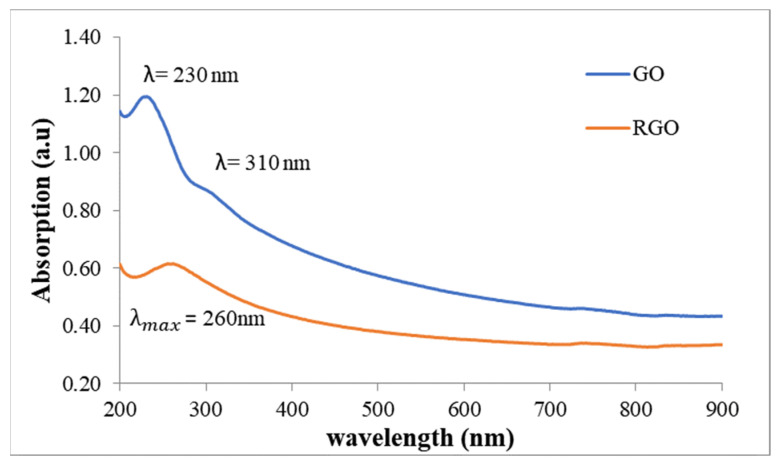
Absorption of pure GO and rGO in chloroform solvent.

As shown in Figure 6, graphene oxide (GO) had distinctive absorption spectra, with a peak at around 230 nm and a shoulder at roughly 310 nm. Its unusual spectral pattern was due to the presence of oxygen-containing functional groups on the GO surface, such as carboxyl, hydroxyl, and epoxy groups. The π-π* causes the peak at the 230 nm transitions of the C=C and C-C bonds inside the graphene lattice, whereas the n causes the shoulder at the 310 nm-π* transitions of the C-O and C=O bonds within the oxygen-containing functional groups. Lai Q et al. (2012) [43] and Saxena S et al. (2011) [44] reported comparable results. By contrast, rGO exhibited a single absorption peak at 260 nm due to residual oxygen-containing functional groups on its surface, such as hydroxyl groups, as observed by Verástegui-Domnguez L. H. et al. [45].

### 3.3. Absorption of the Polymer under GO and rGO Composites in Chloroform

**Figure 7:**
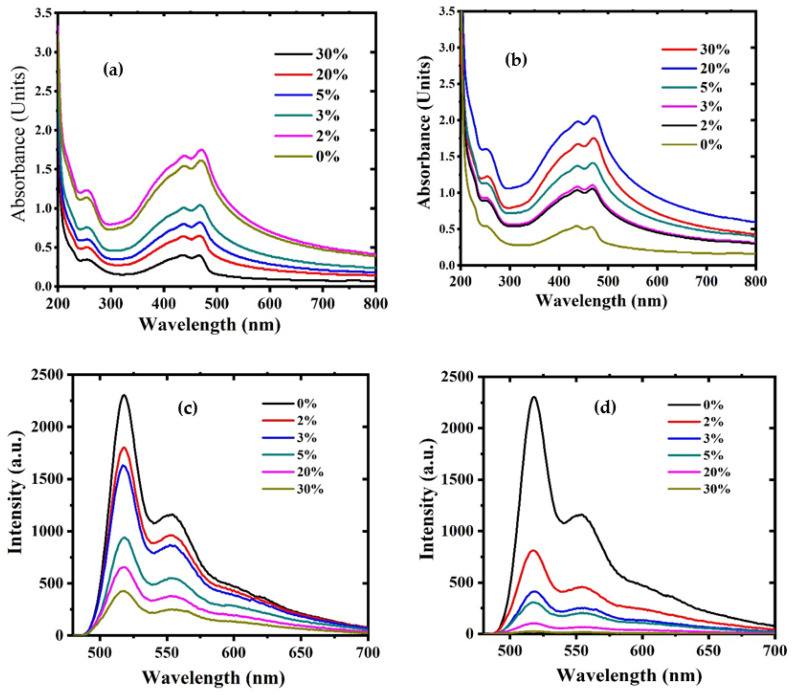
Absorption spectra of (**a**) CP/GO and (**b**) CP/rGO in chloroform. Similarly, the fluorescence of (**c**) CP/GO and (**d**) CP/rGO in chloroform.

Figure 7a,b show the absorption spectra of polymer/GO and polymer/rGO dispersed in chloroform with different weight percentages (2, 3, 5, 20, and 30%). In addition to the GO peak, two clear peaks appeared in the visible region around 440 nm attributed to the n-π* transition and around 480 nm attributed to the π-π* transition. In this study, the concentration of the polymer was higher than the concentration of GO, which prevented the oxygen peak (310 nm in Figure 6) from appearing in the UV-visible spectra [43]. The 20 and 30% samples had a lower wavelength than the other samples at 261 nm and 238 nm for GO and rGO; in effect, the peaks were 438 nm and 470 nm for polymer in GO and 434 nm and 468 nm for polymer in rGO, which might decrease in the delocalized electrons that need higher energy for the electronic transition for these two types of samples.

### 3.4. Fluorescence of CP/GO and CP/rGO Composite in Chloroform

Figure 7c,d show the emission spectra of CP/GO/rGO in chloroform. It is clear from the figures that there are two fluorescence peaks around 540 nm and 560 nm, with excitation at 360 nm. When we added GO and rGO, there was a reduction in the intensity, known as FQ. We observed a reduction in the intensity when we added GO and rGO, but the reduction was more significant when we added rGO. The reason is that FQ occurs in hydrophobic and hydrophilic molecules, but FQ occurs less aggressively in hydrophilic (GO) molecules than hydrophobic (rGO) molecules. Figure 4d shows a broad tail from 575 nm to 650, possibly due to the spectral brooding effect from the inelastic collision of GO and rGO. This is also due to the reabsorption and scattering effect [46]. The rGO quenches fluorescence significantly more than rGO, but this is because GO is less soluble in CP despite sonication due to the low polarity of CP and chloroform. The GO settles faster, and CP becomes more transparent and fluorescent, but rGO and CP form a completely dissolved solution in chloroform. It is well known that GO dissolves more in polar solvents when compared to nonpolar solvents.

### 3.5. Absorption of Polymer/rGO Composite in CHF (Thin Films) 

**Figure 8:**
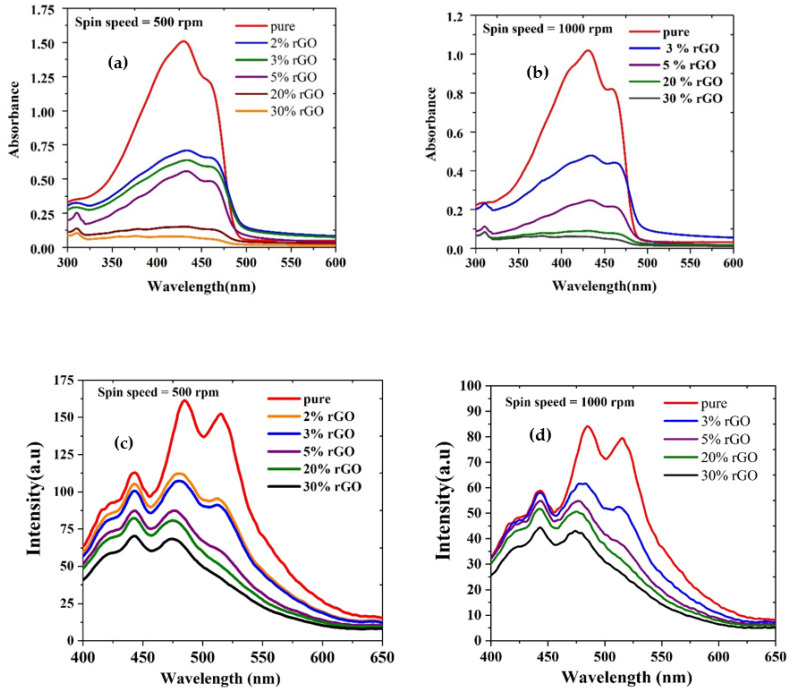
Absorption spectra of pure CP and CP/rGO in chloroform at spin speeds of (**a**) 500 and (**b**) 1000 rpm; and the fluorescence spectra of pure CP and CP/rGO in chloroform that made thin films at spin speeds of (**c**) 500 rpm and (**d**) 1000 rpm.

Figure 8a,b show the absorption spectra of thin films made from CP/rGO of several concentrations (2, 3, 5, 20, and 30% rGO) using two spin speeds—500 rpm and 1000 rpm—named from F1 to F5 (500 rpm) and F6 to F10 (1000 rpm). The respective thicknesses were measured using a profilometer and are listed in Table 1. In general, the addition of rGO did not have a profound effect on the thickness of films compared to spin speed. In addition to the rGO peak, two clear peaks appeared in the visible region around 431 nm attributed to the n-π* transition and around 458 nm attributed to π-π* transition (500 rpm–1000 rpm). The amplitude of the peaks decreased as the concentration of the rGO increased, which showed the successful reduction of GO.

### 3.6. Absorption of Polymer/rGO Composite in CHF (Thin Films)

Figure 8a depicts the absorption spectra of CP/rGO thin films (F1–F5), spin speed = 500 rpm, which demonstrates a decrease in absorption as the concentration of rGO in the polymer increases. This phenomenon could be attributed to changes in the film’s morphology and roughness resulting from the addition of rGO. [28]. Figure 8b depicts the absorption spectra of CP/rGO thin films (F6–F10, spin speed = 1000 rpm), which exhibited a behavior similar to that of the 500 rpm films. The thickness and absorbance of the film depended on the spin speed, which decreased as the speed increased. The presence of rGO in the polymer was evident in the absorption spectra, as an increase in the concentration of rGO resulted in reduced absorption. This decrease could be caused by changes in the film morphology and roughness resulting from adding rGO [28]. 

### 3.7. Fluorescence of Polymer/rGO Composite in CHF (Thin Films)

Figure 8c,d (F6–F10) show the fluorescence spectra of films spin-coated at different speeds, 500 (F1–F5) and 1000 rpm, respectively. Both spectra showed three peaks: the one at 443 nm could be attributed to weak fluorescence/scattering from rGO, the one around 484 nm from the vibrational band (0–1), and last one, around 515 nm, from the (0–2) vibrational band. Further, the fluorescence was increasingly quenched for increased concentrations of rGO. We observed that a reduction in the intensity when adding rGO occurs in the molecules due to the increased FRET (nonradiative) between rGO and CP [42] due to the proximity of the FMOs of both. This is an excellent indication that this device could be designed as a solar energy harvester. 

## 4. Laser Properties

### 4.1. Laser-Induced Fluorescence (LIF) Spectra

**Figure 9:**
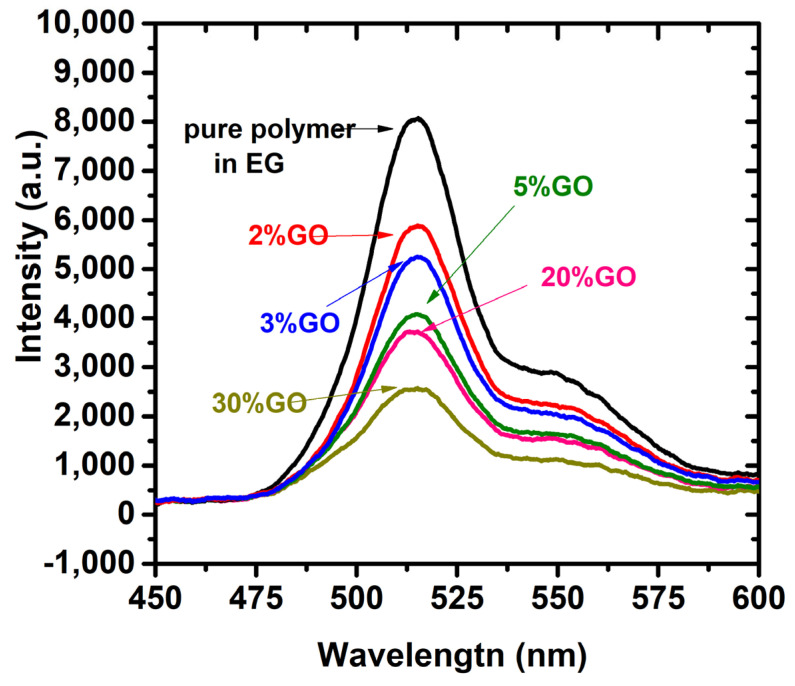
LIF spectra of CP/GO in chloroform.

The LIF spectra of CP/GO in chloroform were measured at a low pump energy of 3 mJ. GO’s FQ and the polymer’s poor solubility in chloroform prevented ASE. This experiment demonstrated that the polymer’s major vibrational bands 0–0, 0–1, and 0–2 correspond to peaks at 450 nm and 514 nm and a shoulder from 525 to 565 nm (Figure 9).

We observed a spectral broadening from 31 nm to 42 nm for the increasing concentration of rGO, as shown in Table S2. The spectral broadening was minimal, up to 10%. However, concentrations above 20 to 30% of rGO induced a significant spectral broadening (>42 nm) and FQ (20% of initial). 

### 4.2. The ASE of CP/rGO in Chloroform and Thin Films

**Figure 10:**
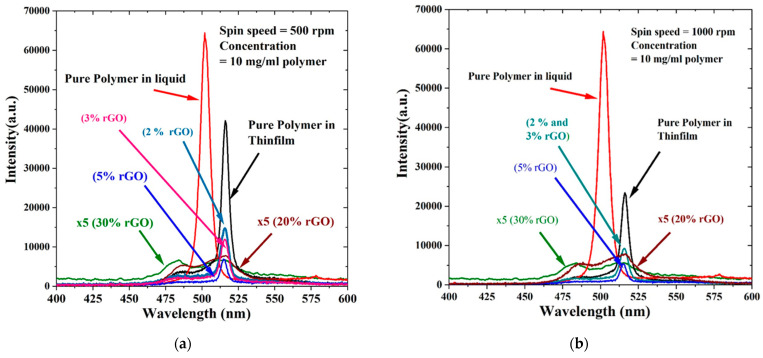
LIF and ASE spectra of CP/rGO in chloroform for thin films of (**a**) 500 rpm and (**b**) 1000 rpm.

Figure 10a illustrates the ASE spectra of CP/rGO thin-film samples F1 to F5 and compares them to the ASEs from CP/rGO in a chloroform solution. The peak wavelength was 15 nm redshifted (the peak in the liquid at 501 nm and in the film at 516 nm). This redshift could be attributed to the reabsorption caused by conformation, tight binding of macromolecules, and damping of lower vibration transitions, and the only allowed transition was 0–2. As the concentration of rGO increased, the quenching of ASEs from the thin film increased. However, ASEs were not completely destroyed until they reached 15%, beyond which quenching and spectral branding became dominant, producing low-intensity and broad spectral fluorescence. The film made at 1000 rpm (F6) showed 55% ASE intensity compared to the 500 rpm film due to the reduced thickness of the film (from 305 nm to 208 nm). ASEs were achieved for concentrations of 2, 3, and 5% but with very low intensity. ASEs were quenched at 20 and 30%, and only LIF was produced. The vibrational transition band 0–2 was dominant with the 20% rGO film (F9), while the primary vibrational transition 0–1 was pronounced for the 30% rGO film (F10) due to the increase in rGO in the polymer films. This reduced LIF intensity is due to reabsorption, and the redshift is due to the vibration-damping effect. As rGO increases the space between polymer chains, the primary vibrational level oscillates, leading to FQ [47]. 

Figure 10b exhibits the LIF and ASE spectra of thin films made from CP/rGO in chloroform dropped and spun at 1000 rpm, and it also compares the ASEs of the pure solution. The results are almost the same as those in Figure 10a, except for a slight increase in the full width at half maximum (FWHM) around 1 nm and a reduction in intensity of about half compared to films F1–F5. Higher speeds above 1000 rpm did not produce uniform films at higher concentrations of rGO. The ASEs were not quenched up to 5%. Hence, by controlling the amount of rGO in the solution and films, we can design new electrically or optically pumped optoelectronic devices with a tunable wavelength, selective charge generation, and intensity enhancement or control in various devices, such as solar cells (high GO and rGO content) and tunable organic light-emitting diodes (OLEDs) (at low GO and rGO concentrations) [48]. 

### 4.3. Time-Resolved Spectroscopy

**Figure 11:**
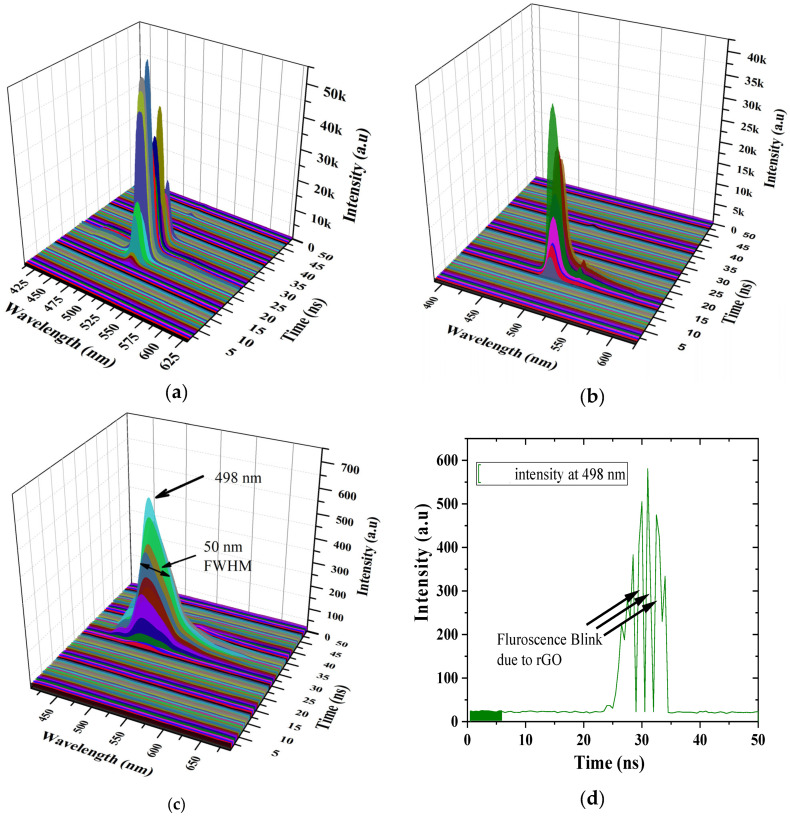
TRS spectra (x = wavelength (nm), y = intensity (a.u), and z = time (ns)) of (**a**) CP/(rGO at 5%), (**b**) CP/(rGO at 20%), (**c**) CP/(rGO at 30%), and (**d**) the Z-slice at wavelength 498 nm.

Figure 11a–c show the different concentrations of rGO from 5 to 30%. This shows that rGO induced the blinking of ASEs or fluorescence in the polymer solution even at 5%, but the spectral width (FWHM) did not change until 10%. However, when the rGO ratio was 20%, the ASE intensity reduced by about 50%, with a slight increase in FWHM (12 nm). This is due to increased quenching by promoting nonradiative decay. When the rGO ratio was increased to 30%, the spectral width was 50 nm, and the intensity was only 1%. Figure 11d shows the Z-slice at the peak wavelength of 498 nm (blue-shifted from 506 nm to 498 nm), showing the complete switch-offs (blinks) at three different time intervals: 28.5 ns, 30.5 ns, and 32.5 ns. This is the direct evidence of ASE blinking; this could occur at different times as each frame is collected after three pumps at each time frame (freeze) for 50 ns at an interval of 0.5 ns [18,49]. 

## 5. Sensor Design

We designed a simple paper-based sensor to demonstrate the composites’ sensing capability. 

**Figure 12:**
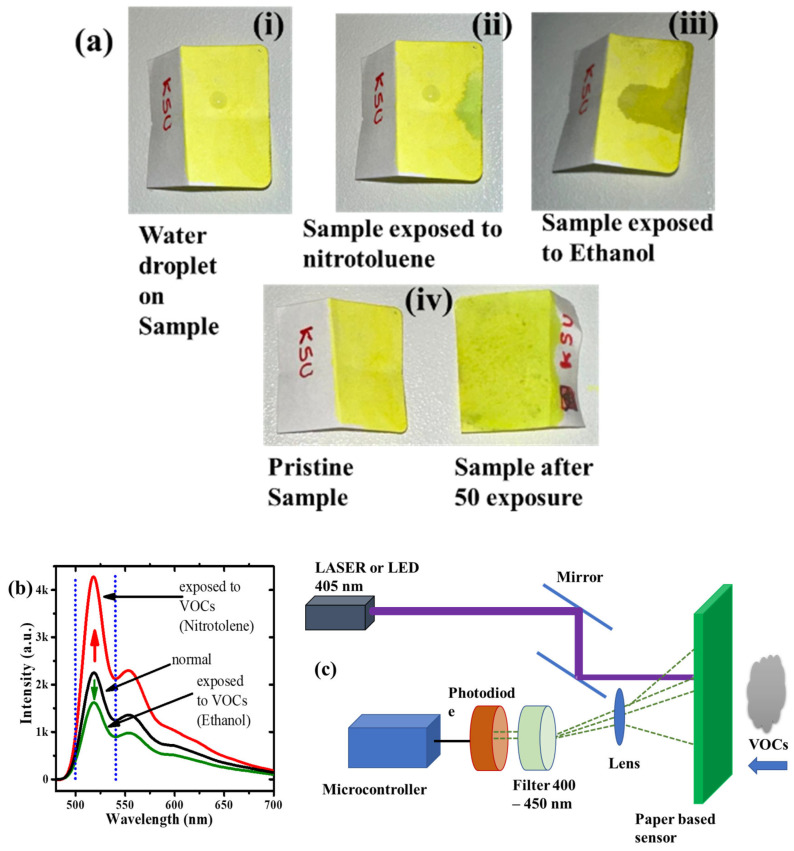
(**a**) Photograph of paper-based volatile organic compounds (VOCs), (**i**) water droplets, (**ii**) nitrotoluene, (**iii**) ethanol, and (**iv**) fresh and exposed samples. (**b**) Fluorescence spectra of the sample under NT and ethanol exposure. (**c**) Schematic of the proposed sensor device.

We have designed a paper-based sensor to detect VOCs using the rGO/CP composites. It was tested as an explosive (nitrotoluene) and alcohol (ethanol) sensor. The best-performing concentration was 10% rGO/CP. The liquid composite was drop-coated on the paper. After drying, the paper became hydrophobic, as shown in Figure 12a. When the sensor sample is exposed to nitrotoluene (NT), the aerosol microdroplets latch onto the surface sensor and locally dissolve the polymer and rGO, dramatically improving the fluorescence of the sensor, visible to the naked eye (as shown in Figure 12a) and measurable using a spectrometer. Figure 12b shows the fluorescence spectra of different VOCs, where the intensity is an arbitrary unit, and the VOCs could be identified by comparing the intensity ratios. Interestingly, we were exposed to ethanol attached to the surface, which increased the fluorescence quenching and was measurable with a spectrometer. We propose designing a simple sensor to detect explosives and alcohol, as in Figure 12c. A 405 nm laser is used to illuminate the sample, and depending on VOCs, the fluorescence is fed to the photodiode through a spectral bandpass filter (500–550 nm). Ethanol can be detected with decreased light output from the sample. When the sample was exposed to NT, it increased the fluorescence output. The sample is reusable after a time ≥30 s; it produced good results up to 100 exposures. The sample can be replaced, and the devices can work again. The device’s output signal is fed to a microcontroller and can be further connected to a display or any connected devices to display the result. 

## 6. Conclusions

We investigated the effect of adding GO and rGO on the polymer PDVF-co-MEH-PV. A preliminary investigation showed that polar solvents were incompatible with the polymer, and GO was incompatible with nonpolar solvents. Chloroform was the best combination solvent for rGO and PDVF-co-MEH-PV to form a homogeneous solution. Hence, we furthered the investigations with chloroform. The results showed that GO was quenched more when compared to rGO, primarily due to the incompatibility between CP and GO. We also found that upon high-energy optical pumping, the rGO/PDVF-co-MEH-PV combination produced ASEs with a narrow spectra peak width of <12 nm (FWHM) for a concentration ratio up to 10%. However, at 20% wt of rGO, the spectra were broad (41 nm), the intensity was dramatically quenched (1% that of pure), and the directionality was lost entirely. The ASEs of thin films were at least 15 nm redshifted compared to pure PDVF-co-MEH-PV in the chloroform liquid, and the ASEs arose from the 0–2 vibrational mode. The simulation showed that the monomer units had two donor sites (oxygens), and rGO has many acceptor sites that readily transfer the excited electrons to the ground, resulting in fluorescence and ASE quenching. However, the rGO/PDVF-co-MEH-PV polymer produced ASEs up to a reasonably high concentration of rGO (10%), showing the potential of its use as an optically and electrically pumped thin-film laser. The fact that the rGO/PDVF-co-MEH-PV is compatible with chloroform can make it much easier to fabricate optoelectronic devices via a simple spin coating process. We also designed a paper-based sensor and proposed a cheap, reusable, and multifunctional instrumentation to detect ethanol and nitrotoluene. The designed sensors reliably reproduced the results of up to 100 tests.

## Figures and Tables

**Table 1:** Thickness of CP/rGO films for spin speeds of 500 and 1000.

Film Name	Speed (rpm)	Concentration Ratio (%)	Thickness (nm)
F1	500	2%	360 nm
F2	500	3%	357 nm
F3	500	5%	358 nm
F4	500	20%	342 nm
F5	500	30%	320 nm
F6	1000	2%	208 nm
F7	1000	3%	210 nm
F8	1000	5%	195 nm
F9	1000	20%	185 nm
F10	1000	30%	181 nm

## Data Availability

Data are available with journal editor and with permission of authors it is for reasonable request.

## Supplementary Materials

The following supporting information can be downloaded at: https://www.mdpi.com/article/10.3390/polym15234572/s1, Figure S1: Dipole moment calculation; Figure S2: FMO of GO and rGO (approximated) in CHF; Figure S3: rGO orbital values in Hartees and orbital numbers; Table S1: vibrational transitions, its contributions, and the oscillator strengths of the first excited state of rGO from the simulation; Table S2: the ASE’s spectral broadening of CP with respect of rGO.
