# Supplementary material for: Effects of Graphene Oxide (GO) and Reduced Graphene Oxide (rGO) on Green-Emitting Conjugated Copolymer’s Optical and Laser Properties Using Simulation and Experimental Studies
Source: Polymers (Basel). 2023 Nov 29;15(23):4572. doi: 10.3390/polym15234572 (PMC10708564; doi:10.3390/polym15234572)
Supplement: Supplementary file 1 [file polymers-15-04572-s001.zip › polymers-2629007-supplementary.pdf]

Supplementary Files

Effects of Graphene Oxide (GO) and Reduced Graphene Oxide (rGO) on Green-Emitting Conjugated Copolymer's Optical and Laser Properties Using Simulation and Experimental Studies

Saradh Prasad *, Raya H. Alhandel, Nassar N. Asemi and Mohamad S. AlSalhi

Research Chair on Laser Diagnosis of Cancers, Department of Physics and Astronomy, College of Science, P.O. Box 2455, King Saud University, Riyadh 11451, Saudi Arabia; nassarasemi@gmail.com (N.N.A.); malsalhi@ksu.edu.sa (M.S.A.)
* Correspondence: srajendra@ksu.edu.sa; Tel.:+966-50-101-5786

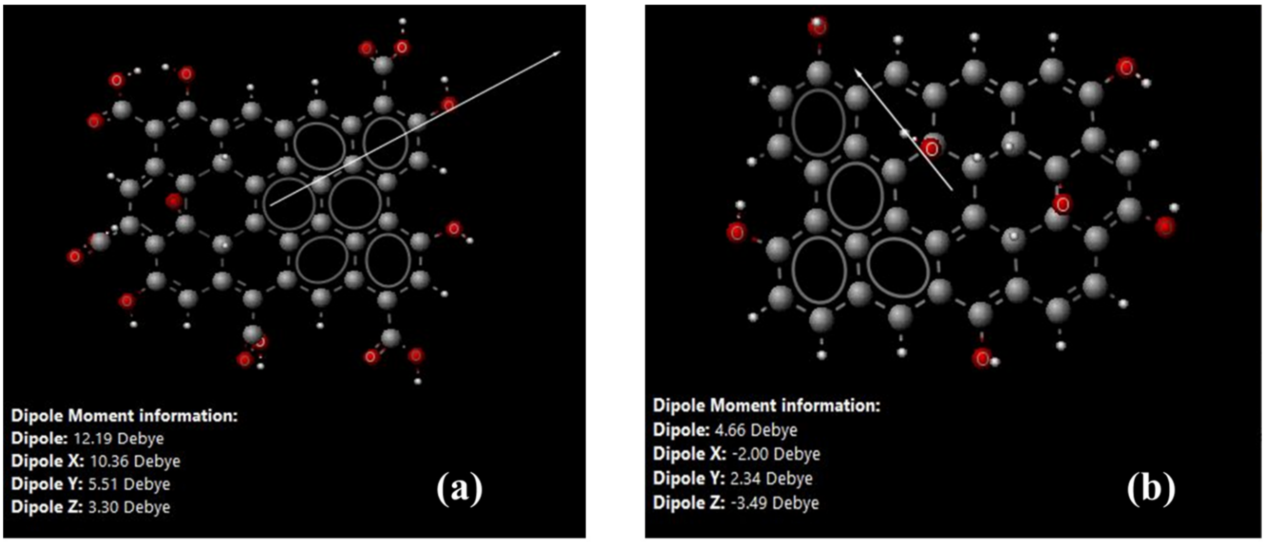

Dipole moment of (a) GO and (b) rGO

Figure S1 Dipole moment calculation

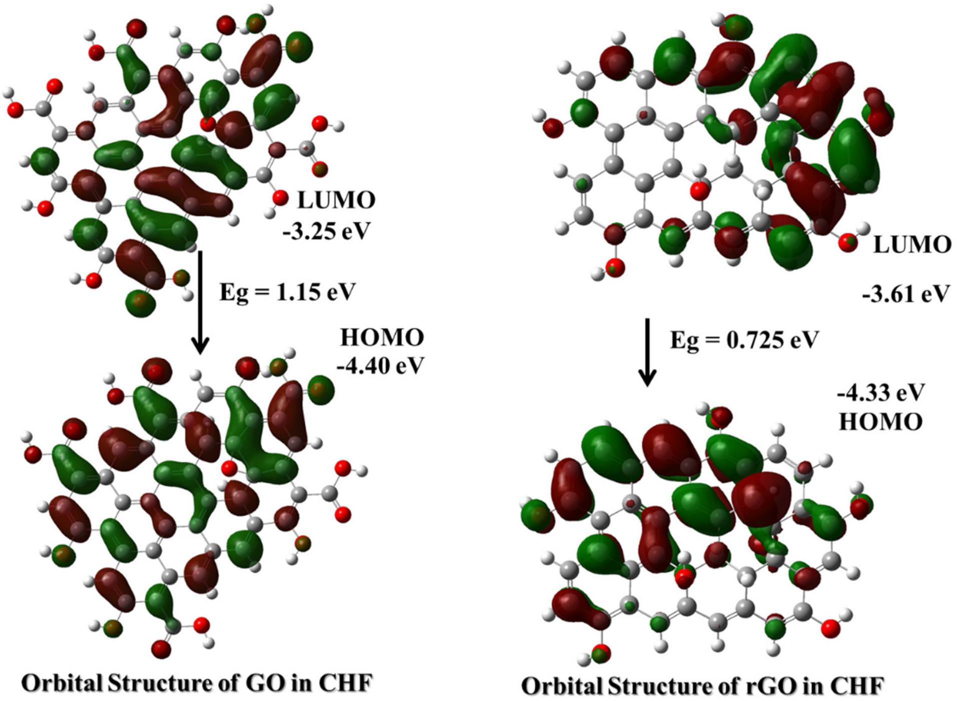

Figure S2 FMO of GO and rGO (approximated) in CHF

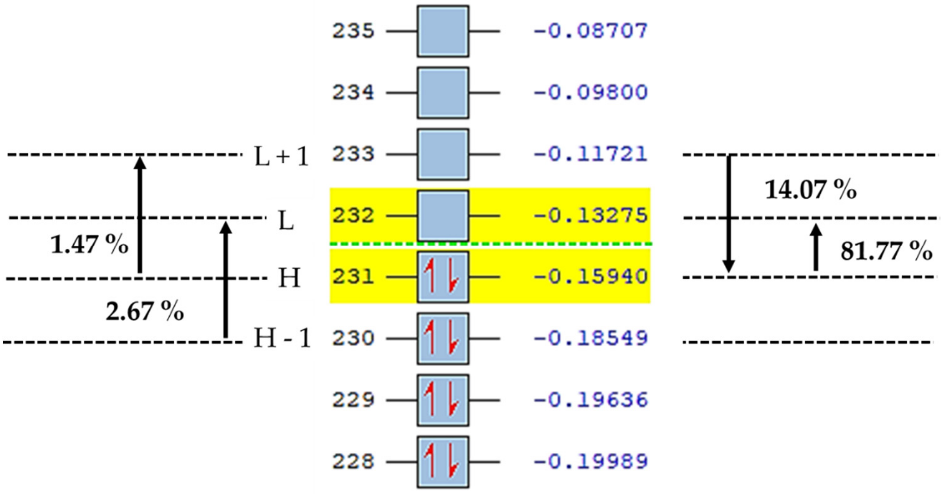

Figure S3 rGO orbital values in Hartees and orbital numbers.

Table S1, vibrational transitions, its contributions, and the oscillator strengths of the first excited state of rGO from the simulation

Excited States	Contributions	Oscillator strengths
H → L	81.77%	0.74734
(H-1) → L	2.67%	0.13519
H → (L + 1)	1.47%	0.10034
(L + 1) → H	14.07%	0.31002

Table S2, the ASE's spectral broadening of CP with respect of rGO

**rGO (Wt%) in CHF**	**FWHM start**	**FWHM end**	**FWHM**
	**(nm)**		
0%	499 **nm**	530 **nm**	31 **nm**
2%	499 **nm**	532 **nm**	33 **nm**
3%	499 **nm**	532 **nm**	33 **nm**
5%	497 **nm**	534 **nm**	37 **nm**
20%	496 **nm**	535 **nm**	39 **nm**
30%	493 **nm**	535 **nm**	42 **nm**
